# Abnormal Serum Iron-Status Indicator Changes in Amyotrophic Lateral Sclerosis (ALS) Patients: A Meta-Analysis

**DOI:** 10.3389/fneur.2020.00380

**Published:** 2020-05-20

**Authors:** Lan Wang, Chunyu Li, Xueping Chen, Shuying Li, Huifang Shang

**Affiliations:** Department of Neurology, West China Hospital, Sichuan University, Chengdu, China

**Keywords:** amyotrophic lateral sclerosis, iron metabolism, ferritin, transferrin, iron

## Abstract

**Background:** In recent years, the role of iron metabolism in amyotrophic lateral sclerosis (ALS) attracts more and more attention, and some studies have focused on the link between abnormal serum iron indicators and ALS. However, there are still big conflicts and inconsistency among different studies. To study the possible relationship between ALS and disturbed iron metabolism, we conducted this meta-analysis to conclude characteristics of abnormal serum iron-status indicator changes in ALS patients.

**Methods:** We searched and screened main databases, including the PubMed, Embase, and Cochrane Library, to find studies related to the association between iron metabolism and ALS. The Revman 5.3 software was used to conduct meta-analysis.

**Results:** Eleven studies were finally included in our analysis, composed of 1,599 ALS patients and 1,255 controls in total. The results showed that the ferritin level was much higher in ALS patients compared with controls (MD = 70.48, 95% CI [51.41, 89.55], *p* < 0.00001), and the transferrin level was decreased in ALS patients compared with controls (SMD = −0.28, 95% CI [−0.38, −0.18], *p* < 0.00001), while there was no statistical difference in iron levels (SMD = 0.48, 95% CI [−0.07, 1.03], *p* = 0.09) between ALS patients and controls.

**Conclusions:** Our research finds unusual changes in several indicators representing iron status, which suggest possible iron metabolism abnormalities in ALS patients. That may provide evidence for the link between iron metabolism and the pathogenesis of ALS.

## Introduction

Amyotrophic lateral sclerosis (ALS) is a fatal neurodegenerative disorder with both upper and lower motor neurons affected (1). As an adult-onset disease, ALS has only 3–5 years of median survival time after symptom onset, which brings huge burden to both the ALS families and society. Existing evidences have suggested many hypotheses explaining the neurodegeneration of ALS, such as oxidative stress, abnormal neuroinflammation, gene mutations, metabolic dysfunction, and metal toxicity (2–4). However, the pathogenesis of the rare disorder remains unclear. Exploration of pathogenesis processes and biomarkers specific to ALS may benefit the early diagnosis or prognosis prediction and suggest targets for novel therapies.

Recently, abnormal iron metabolism has been proved to be related to neurodegenerative disorders, including ALS (5–8). Iron, as one necessary microelement of human, participates in many biological processes of human brain, such as DNA and myelin synthesis, oxygen transportation, and iron homoeostasis, and is pivotal to maintain normal brain function (8). Iron dyshomeostasis affects pathways such as oxidative stress, which was reported to be implicated in ALS a long time ago (4, 9–11). Furthermore, iron serum-status indicators, including serum iron, ferritin, and transferrin, also have been studied in ALS, and these indicators may function as potential ALS biomarkers (12, 13). For example, in a retrospective analysis, Nadjar et al. (12) showed that serum ferritin levels were higher and serum transferrin levels were lower in ALS patients than controls, and the ferritin level was associated with ALS patients' survival time. Veyrat-Durebex et al. (14) found that serum iron was higher in ALS patients compared with controls, while other studies showed there were no differences between patients and controls (12, 15). Despite accumulative evidence showing that abnormal iron metabolism is closely related to ALS pathogenesis and there are some studies linking the serum iron indicators (iron ion, ferritin, and transferrin) to ALS diagnosis, progression, and even survival, there are big conflicts and inconsistency among different studies.

Here, we conducted a meta-analysis to conclude the characteristics of serum iron indicators in ALS patients, which may provide a favorable foundation for the coming researches and the neurological physicians.

## Methods

This study was conducted according to the “Preferred Reporting Items for Systematic Reviews and Meta-analyses” (PRISMA) guidelines (16).

### Search Strategies

Several main databases, including the PubMed, Embase, and Cochrane Library, and library collections were searched to find studies related to the association between iron serum-status indicators and ALS before 31st March 2019. Thesaurus plus free words were used for searching. The specific search strategy of PubMed is shown as follows:

(“Sclerosis, Amyotrophic Lateral” OR “Charcot Disease” OR “Lou Gehrig Disease” OR “Disease, Lou-Gehrigs” OR “Gehrig Disease” OR “ALS” OR “Guam Disease” OR “Amyotrophic Lateral Sclerosis”[Mesh]) AND (“Serotransferrin” OR “Siderophilin” OR “beta-1 Metal-Binding Globulin” OR “Globulin, beta-1 Metal-Binding” OR “Isotransferrin” OR “Metal-Binding Globulin, beta-1” OR “beta 1 Metal Binding Globulin” OR “tau Transferrin” OR “beta 2-Transferrin” OR “Monoferric Transferrins” OR “Transferrin"[Mesh]” OR “Ferritin” OR “Isoferritin” OR “Basic Isoferritin” OR “Isoferritin, Basic” OR “Ferritins”[Mesh] OR “Ferric Compounds” OR “Ferrous Compounds” OR “Hemosiderin” OR “Hemosiderosis” OR “Iron Isotopes” OR “Siderosis” OR “Iron”[Mesh] OR “iron-binding protein” OR “iron metabolism” OR “iron status” OR “iron intake”).

### Inclusion and Exclusion Criteria

The included studies must meet the following criteria: (1) patients with diagnoses of ALS according to the El Escorial diagnostic criteria, with definite, probable, laboratory probable, or possible, or from a large motor neuron disease research center with guaranteed quality; (2) evaluating the indicator level of iron metabolism; and (3) detailed information, such as patients' ethnicity, nationality, and duration of disease were extracted from all the included studies. Exclusion criteria were as follows: (1) duplicate reports; (2) review or abstract of conference or animal studies; (3) necessary data were not available; (4) non-English literature; and (5) different studies from the same population.

### Data Extraction

Literature screening and data extraction were processed by two researchers independently, and there was a cross-check eventually. In case of disagreement, a third party was consulted to help judge. Whether the controversial one was included or not depended on the result of discussion. The main contents of the data extraction included the following: (1) basic information of the included researches: researches' topic, author, country, and time of publication; (2) the baseline characteristics of the research objects: the sample size of each group, sex ratio, disease status, etc.; (3) observation indicators: mean and standard deviation (SD) of serum transferrin, ferritin, and iron levels; and (4) key elements to conduct risk of bias.

### Quality Assessment

The Newcastle–Ottawa Scale (NOS) (17) was used to carry out quality of included case–control and cohort studies. This scale used a star system's quantitative principle, out of nine stars. The quality of cross-sectional studies included was assessed using an 11-item checklist, which was recommended by the Agency for Healthcare Research and Quality (AHRQ) (18). Article quality was assessed as follows: low quality = 0–3; moderate quality = 4–7; high quality = 8–11.

### Data Analysis

The Revman 5.3 software was used to do meta-analysis. When comparing serum iron level, ferritin level, and transferrin level between ALS patients and controls, mean difference (MD) or standard mean difference (SMD) is chosen as the effect indicator, according to the Cochrane handbook, and each effect quantity gives its point estimate and 95% CI. The heterogeneity analysis was assessed by χ^2^ statistic and the value of *I*^2^; meanwhile, if the *I*^2^ <25% and *p* > 0.1, the included studies were mild heterogeneous, and the fixed effect model was used. Otherwise, the random effect model was used to calculate the combined estimate when the heterogeneity was moderate (*I*^2^ > 50%) or obvious (*I*^2^ > 75%). Publication bias was assessed by funnel plot and Egger's test. To evaluate the influence of each individual study on the pooled estimate, sensitivity analysis was conducted by omitting each study one by one.

## Results

### Characteristics of the Included Studies

**Figure 1:**
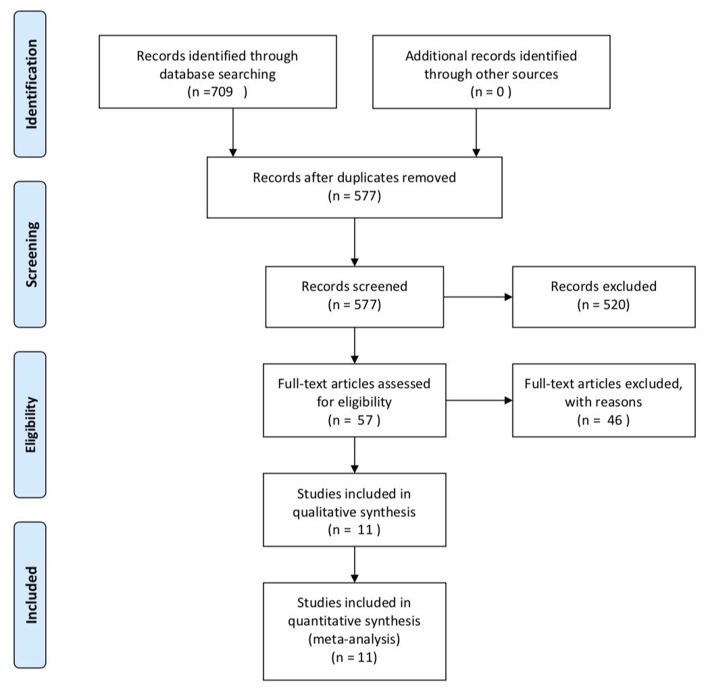
Specific process of research selection.

A total of 709 literatures were identified after searching the database. There were 577 papers after removing duplicate documents and 520 papers among them were further removed after reading titles and abstracts. Then, 57 papers were under full-text review. Finally, 11 researches were enrolled, including five cohort studies and six cross-sectional studies (12–15, 19–25). The specific process of selection is shown in Figure 1. These 11 studies included 1,599 ALS patients and 1,255 controls in total. Most studies use the Revised El Escorial criteria (1) to determine patients and every study included different diagnosis-level patient. Most studies choose healthy controls. Five studies were conducted in Europe, while three were conducted in China, one in Japan, and two in America. In the NOS part, every study's score was over six stars, and in the AHRQ assessment, all studies' scores were over 5, showing that they were all of moderate quality to high quality. All the details are shown in Table 1.

**Table 1:** General characteristics of included studies.

	**Research type**	**Location**	**ALS/Control**	**Age (ALS/Control)**	**Diagnostic category**	**ALS definition**	**Disease duration (months)**	**ALSFRS-R**	**Quality assessment**	**Ferritin assay**	**Transferrin assay**	**Iron assay**
Goodall et al. (15)	Cross-sectional	UK	60/44	60/59^a^	NM	NM	NM	NM	5	NM	NM	CMM
Qureshi et al. (23)	Cross-sectional	USA	30/30	54.4/52.5^b^	NM	EEC	NM	31.9 (5.7)^c^	8	EIA	–	–
Yu et al. (25)	Cross-sectional	China	24/38	51.96/53.32^b^	def/pro	EEC	6–36^c^	20–42^d^	8	CLM	–	CMM
Ikeda et al. (22)	Cross-sectional	Japan	92/92	58.8/59.2^b^	def/pro	REEC	23.7 (21.3)^c^	40.3 (3.9)^c^	9	EIA	NIA	NM
Mitchell et al. (21)	Cross-sectional	USA	29/36	61/54^a^	def/pro/lab-pro/pos	REEC	19 (12-28)^f^	NM	7	–	NM	–
Blasco et al. (20)	Cross-sectional	France	9/10	65.7/65.4^b^	def/pro	REEC	NM	41.3 (3.7)^c^	8	–	–	NM
Nadjar et al. (12)	Cohort	France	694/297	61.85/48.99^b^	def/pro/lab-pro	REEC	NM	NM	8	NIA	NIA	CMM
Veyrat-Durebex et al. (14)	Cohort	France	104/145	67.6/68.4^b^	def/pro	REEC	41.2^b^	37 (7)^c^	8	CLM	NIA	CMM
Zheng et al. (19)	Cohort	China	54/46	53.85/51.26^b^	def/pro	REEC	14.99 (1.82)^e^	34.31 (0.93)^e^	7	–	ELISA	–
Lu et al. (24)	Cohort	UK	95/88	66.8/59.1^a^	def/pro/lab-pro/pos	REEC	22.4 (12.5–32.2)^f^	39 (31-43)^f^	8	EIA	–	–
Sun et al. (13)	Cohort	China	435/431	55.33/54.85^b^	def/pro/pos	REEC	NM	NM	7	EIA	NIA	EIA

*ALS, amyotrophic lateral sclerosis; BMI, body mass index; EEC, El Escorial criteria; REEC, Revised El Escorial criteria; def, definite; pro, probable; lab-pro, laboratory probable; pos, possible; NM, not mentioned; NIA, nephelometric immunoassay; EIA, electrochemiluminescence immunoassay; CLM, chemiluminescence method; CMM, colorimetric method; ^a^median, ^b^mean, ^c^mean (SD), ^d^minimum–maximum, ^e^mean (SE), ^f^median (interquartile range)*.

### Results of Meta-Analysis

#### Comparing Ferritin Level Between ALS Patients and Controls

**Figure 2:**
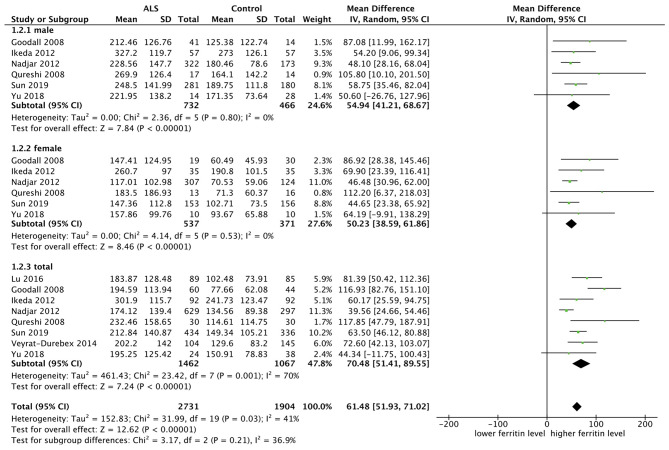
The result showed that ferritin was higher in ALS patients, no matter in the male subgroup (MD = 54.94, 95% CI [41.21, 68.67], *p* < 0.00001) or female subgroup (MD = 50.23, 95% CI [38.59, 61.86], *p* < 0.00001). The result in total showed the same result (MD = 70.48, 95% CI [51.41, 89.55], *p* < 0.00001).

Eight studies included ferritin as one indicator and there was a moderate heterogeneity between them (*p* = 0.001, *I*^2^ = 70%), so we chose the random model. Mean difference (MD) was chosen since effect indicator and the measurement methods of included studies were basically consistent. Six studies carried out subgroup analysis by sex. The result showed that ferritin was higher in ALS patients, no matter in the male subgroup (MD = 54.94, 95% CI [41.21, 68.67], *p* < 0.00001) or female subgroup (MD = 50.23, 95% CI [38.59, 61.86], *p* < 0.00001). The result in total ALS group showed the same (MD = 70.48, 95% CI [51.41, 89.55], *p* < 0.00001). In the subgroup analysis, the heterogeneity between studies was significantly reduced, as shown in the male subgroup (*p* = 0.80, *I*^2^ = 0%) and female subgroup (*p* = 0.53 *I*^2^ = 0%), suggesting that the sex could be one of the sources of heterogeneity. The forest plot is shown in Figure 2.

#### Comparing Iron Level Between ALS Patients and Controls

**Figure 3:**
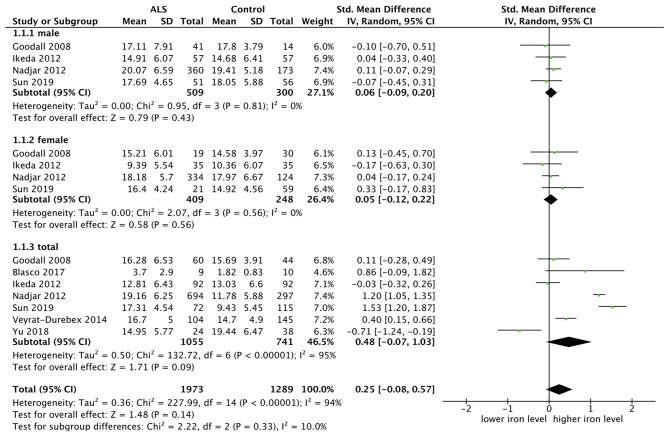
The result showed that there was no statistical difference of iron level both in the male subgroup (SMD = 0.06, 95% CI [−0.09, 0.20], *p* = 0.43) and in the female subgroup (SMD = −0.05, 95% CI [−0.12, 0.22], *p* = 0.56). The result in the total sample group showed the same (SMD = 0.48, 95% CI [−0.07, 1.03], *p* = 0.09).

Seven researches were included for analysis of iron level, and we chose the random model since there was an obvious heterogeneity (*p* < 0.00001, *I*^2^ = 95%). SMD was chosen because the measurement methods of two included studies cannot be decided and the rest were not completely same. Four studies carried out subgroup analysis by sex. The result showed that there was no statistical difference in iron level both in the male subgroup (SMD = 0.06, 95% CI [−0.09, 0.20], *p* = 0.43) and in the female subgroup (SMD = −0.05, 95% CI [−0.12, 0.22], *p* = 0.56). The result in the total group showed the same (SMD = 0.48, 95% CI [−0.07, 1.03], *p* = 0.09). In the subgroup analysis, the heterogeneity between studies was significantly reduced, as shown in the male subgroup (*p* = 0.81, *I*^2^ = 0%) and female subgroup (*p* = 0.56, *I*^2^ = 0%), suggesting that the sex could be one of the sources of heterogeneity. The forest plot is shown as Figure 3.

#### Comparing Transferrin Level Between ALS Patients and Controls

**Figure 4:**
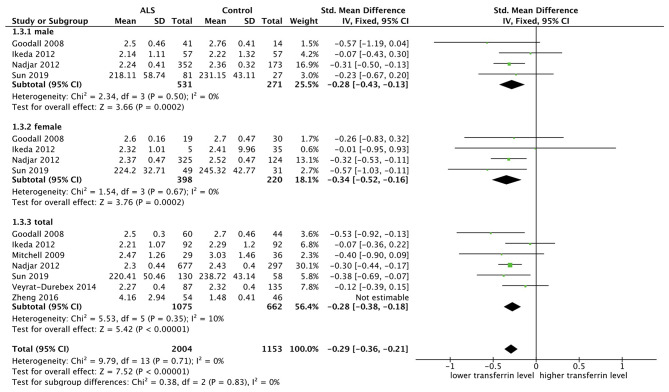
The result showed in the male subgroup (SMD = −0.28, 95% CI [−0.43, −0.13], *p* = 0.0002) and female subgroup (SMD = −0.34, 95% CI [−0.52, −0.16], *p* = 0.0002) that the transferrin level was lower in ALS patients, and the results in total showed the same (SMD = −0.28, 95% CI [−0.38, −0.18], *p* < 0.00001).

Seven researches were included and fixed model was used since there was no substantial heterogeneity (*p* = 0.35, *I*^2^ = 10%). SMD was chosen because two studies did not provide the specific measurement methods. The transferrin level was lower in the male subgroup (SMD = −0.28, 95% CI [−0.43, −0.13], *p* = 0.0002) and in the female subgroup (SMD = −0.34, 95% CI [−0.52, −0.16], *p* = 0.0002), in total ALS group (SMD = −0.28, 95% CI [−0.38, −0.18], *p* < 0.00001). The forest plot is shown in Figure 4.

### Sensitivity Analysis

Sensitivity analysis demonstrated that the pooled effect indicators of the three meta-analyses were stable after omitting each study, which suggested that the results were reliable. However, in the analysis of transferrin level, when deleting the research (19), the heterogeneity in total changed from 88 to 10%, prompting that this study has an abnormal interference with the final result. After checking the measurement methods of transferrin, its method was totally inconsistent with others, so we excluded the study in the final calculation.

### Publication Bias

**Figure 5:**
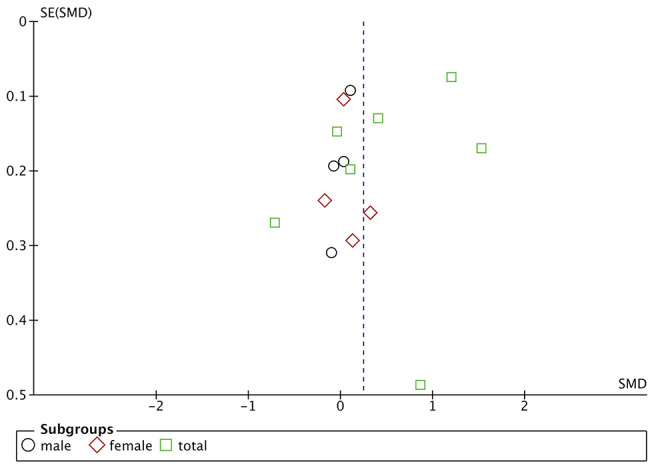
Publication bias assessment of the studies included iron level as one indicator. SE, standard error.

**Figure 6 F6:**
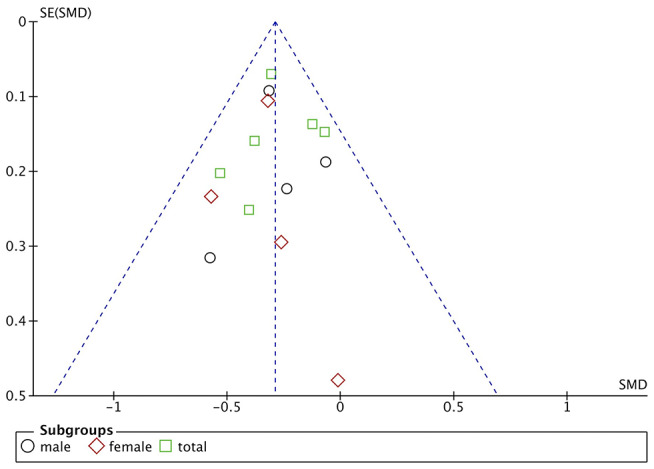
Publication bias assessment of the studies included transferrin level as one indicator.

**Figure 7:**
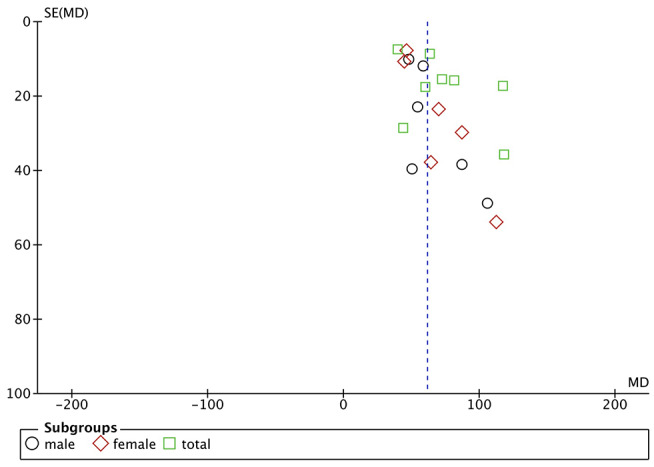
Publication bias assessment of the studies included ferritin level as one indicator.

Funnel plots are drawn using RevMan 5.3 software for the included studies. The funnel plots were not completely symmetrical and funnel shaped, indicating possible publication bias (Figures 5–7). For quantitative analysis, Egger's test was processed through Stata 15.1 software. Ultimately, Egger's test showed that there was no significant publication bias. For studies that included transferrin level as one indicator, *p* = 0.429; for iron, *p* = 0.215; for ferritin, *p* = 0.103 (detailed data for Egger's test are shown in Supplementary Materials, Supplementary Figure 1). [Fig F6]

## Discussion

Iron is an indispensable trace element of organisms, which not only is involved in hemoglobin metabolism but also plays an important role in the development and function maintenance of the nervous system (8, 26). Previous studies have shown that excessive and unstable iron can catalyze oxidative action and generate ROS (4, 7, 8), causing cell damage and even inducing cell apoptosis (27, 28), which may be tightly involved in ALS (9–11). However, the changes of the serum iron-status indicators in ALS are still inconsistent and debatable. In the present study, we systematically summarize the changes of serum iron, ferritin, and transferrin in ALS patients using meta-analysis. Through systematic retrieval of the literatures and quality assessment, 11 related studies with high quality were finally included. The majority of the included studies have adjusted for other risk factors and the included studies covered different countries. Thus, this is a meta-analysis based on a group of well-recognized and credible literature. Our results showed that serum ferritin level was higher and transferrin level was lower in ALS patients compared with healthy controls, while there was no statistical difference in iron between ALS patients and healthy controls.

Ferritin is a major storage protein for iron and plays an important role in cellular iron cycle (29). It consists of two different subunits: ferritin heavy chain (H-ferritin) and ferritin light chain (L-ferritin) (7, 8). H-ferritin has the ability to transform iron into a lower active form (Fe^3+^), thus reducing the reserve of unstable Fe^2+^ and the damage of oxidation reaction (7). So ferritin is believed to play an antioxidant role both in physiological and pathological conditions (30, 31). The anti-oxidative activity of ferritin is closely related to oxidative stress, and its expression can be promoted by oxidative stress (12, 31). From this point of view, ferritin may possess a protective role in the process of neurodegenerative disease. In the current study, we found that the ferritin level was statistically higher in ALS patients, which may be a self-response to the state oxidative stress in ALS. However, some studies show that the increased serum ferritin is associated with shorter survival time (12, 24). Moreover, TNF-α and IL-1β, released by activated microglia, could promote astrocytes and microglia secreting ferritin, which couples the ferritin and neuroinflammation (32), while the molecular mechanisms underlining the connection is largely unknown. Therefore, whether the elevated ferritin is a protective or risk factor of disease is still unclear, and further exploration of the molecular mechanism is needed. From our study, ferritin could potentially be used as a screening biomarker of ALS at least.

Transferrin is an important iron transport protein, which is believed could reduce the level of free iron and thus possess a protective effect for neurons in ALS (33, 34). Our study showed that transferrin level was lower in ALS patients than in controls, prompting the idea the lower transferrin was associated with increased risk of developing ALS. Moreover, the serum transferrin level is regulated by homeostatic iron regulator (encoded by *HFE*). The *HFE H63D* gene polymorphism is associated with decreased transferrin in healthy populations (35), while the *H63D HFE* gene polymorphism is considered as a risk factor for ALS (36) and associated with a shorter survival in animal models (37). Even though the effect of *HFE H63D* polymorphism on the survival of ALS patients remains discrepant, some studies suggest that *HFE* genotypes may interact with *SOD1* mutations, which may influence the survival of ALS patients (38–40). Therefore, the downregulation of transferrin may be a toxic intermediate in ALS, which is consistent with our results.

High iron level is usually considered as toxic in neurodegeneration, and iron chelator therapy given in ALS mouse models could effectively reduce the loss of motor neurons and extend the survival period of mice, suggesting that these mice may have higher iron level and the decline of iron level lead to reduced toxic effects of iron accumulation (41, 42). However, we did not find any statistical difference in iron level between ALS patients and control. Considering the obvious heterogeneity of included studies when analyzing iron level, more evidence of relationship between iron levels and ALS patients is needed.

Another aspect worth discussing is nutrition impact on iron-status indicators. As we know, some patients may have swallowing dysfunction as the disease progresses, resulting in rapid decline in nutritional status and causing insufficient iron intake. In the current study, we found that serum ferritin level was higher and transferrin level was lower in ALS patients compared with healthy controls, while no statistical difference in iron was observed between ALS patients and healthy controls, suggesting that the nutrition impact may be subtle on the serum iron-status indicators. What's more, it is noticeable that the iron-status indicators are correlated with ALS patients' disease progression rate. For example, Lu's work suggested that ALS patients with a faster disease progression rate have a trend of higher serum ferritin (24), and some other studies reported that the serum iron-status indicators may be associated with the site of onset (12–15, 25) in ALS. Unfortunately, the data of included studies cannot be combined for quantitative analysis due to different statistical methods and data type.

There are some limitations in our study. There was obvious heterogeneity of included studies when performing a meta-analysis of iron level, while there was mild to moderate heterogeneity in studies of ferritin and transferrin levels, proving the reliability of the changes of ferritin and transferrin in ALS at least. In addition, we found that the burden of the French and Chinese population exceeded that of the other populations in the final analysis and half of the total cohort came from France. However, there was no obvious publication bias and sensitivity analysis showed that the results are stable. Considering there is no research to prove that iron metabolism indicators are related to ethnicity currently, the overrepresentation of two populations may not affect the results. There are some other limitations such as some of the included studies had small samples and inconsistent design, and some studies were cross-sectional study, and we are unable to determine the exact causal relationship between iron metabolism and the development of ALS.

## Conclusion

In summary, serum ferritin levels in patients with ALS are significantly higher and transferrin levels are lower than those in healthy controls, while no statistical difference in the iron level between ALS patients and controls was identified. Further prospective and multi-centered studies of the changes of iron-status indicators and role of iron metabolism in ALS are needed.

## Data Availability Statement

The raw data supporting the conclusions of this article will be made available by the authors, without undue reservation, to any qualified researcher.

## Author Contributions

All authors contributed to manuscript revision, and read and approved the submitted version. LW, CL, and HS contributed to the conception and design of the study. LW and SL were responsible for data collection, collation, and data analysis. LW wrote the first draft of the manuscript. XC and CL supervise the process and provide guidance. CL and HS revised the final manuscript.

## Conflict of Interest

The authors declare that the research was conducted in the absence of any commercial or financial relationships that could be construed as a potential conflict of interest.
