# Supplementary material for: Abnormal Serum Iron-Status Indicator Changes in Amyotrophic Lateral Sclerosis (ALS) Patients: A Meta-Analysis
Source: Front Neurol. 2020 May 20;11:380. doi: 10.3389/fneur.2020.00380 (PMC7251146; doi:10.3389/fneur.2020.00380)
Supplement: Supplementary file 1 [file Data_Sheet_1.DOCX]

Supplementary Material

# Supplementary Figures

#

figure(A)

figure(B)

figure(C)

figure(D)

figure(E)

figure(F)

## Supplementary Figure 1. The results of Egger’s test

figure(A) and figure(B) Egger’s test for studies included iron level as one indicator.

figure(C) and figure(D) Egger’s test for studies included transferrin level as one indicator

figure(E) and figure(F) Egger’s test for studies included ferritin level as one indicator
